# Effects of Resisted Sprint Training and Traditional Power Training on Sprint, Jump, and Balance Performance in Healthy Young Adults: A Randomized Controlled Trial

**DOI:** 10.3389/fphys.2018.00156

**Published:** 2018-03-02

**Authors:** Olaf Prieske, Tom Krüger, Markus Aehle, Erik Bauer, Urs Granacher

**Affiliations:** Division of Training and Movement Sciences, Research Focus Cognition Sciences, University of Potsdam, Potsdam, Germany

**Keywords:** specificity, sprinting, jumping, change-of-direction speed, balance

## Abstract

Power training programs have proved to be effective in improving components of physical fitness such as speed. According to the concept of training specificity, it was postulated that exercises must attempt to closely mimic the demands of the respective activity. When transferring this idea to speed development, the purpose of the present study was to examine the effects of resisted sprint (RST) vs. traditional power training (TPT) on physical fitness in healthy young adults. Thirty-five healthy, physically active adults were randomly assigned to a RST (*n* = 10, 23 ± 3 years), a TPT (*n* = 9, 23 ± 3 years), or a passive control group (*n* = 16, 23 ± 2 years). RST and TPT exercised for 6 weeks with three training sessions/week each lasting 45–60 min. RST comprised frontal and lateral sprint exercises using an expander system with increasing levels of resistance that was attached to a treadmill (h/p/cosmos). TPT included ballistic strength training at 40% of the one-repetition-maximum for the lower limbs (e.g., leg press, knee extensions). Before and after training, sprint (20-m sprint), change-of-direction speed (T-agility test), jump (drop, countermovement jump), and balance performances (Y balance test) were assessed. ANCOVA statistics revealed large main effects of group for 20-m sprint velocity and ground contact time (0.81 ≤ d ≤ 1.00). *Post-hoc* tests showed higher sprint velocity following RST and TPT (0.69 ≤ d ≤ 0.82) when compared to the control group, but no difference between RST and TPT. Pre-to-post changes amounted to 4.5% for RST [90%CI: (−1.1%;10.1%), d = 1.23] and 2.6% for TPT [90%CI: (0.4%;4.8%), d = 1.59]. Additionally, ground contact times during sprinting were shorter following RST and TPT (0.68 ≤ d ≤ 1.09) compared to the control group, but no difference between RST and TPT. Pre-to-post changes amounted to −6.3% for RST [90%CI: (−11.4%;−1.1%), d = 1.45) and −2.7% for TPT [90%CI: (−4.2%;−1.2%), d = 2.36]. Finally, effects for change-of-direction speed, jump, and balance performance varied from small-to-large. The present findings indicate that 6 weeks of RST and TPT produced similar effects on 20-m sprint performance compared with a passive control in healthy and physically active, young adults. However, no training-related effects were found for change-of-direction speed, jump and balance performance. We conclude that both training regimes can be applied for speed development.

## Introduction

Regular physical exercise (e.g., resistance training) defined as a specific subset of physical activity maintains and develops physical fitness, health, and wellness (Sjøgaard et al., 2016; Chieffi et al., 2017). For instance, it has frequently been shown that different types of resistance training (e.g., power training, plyometric training etc.) have the potential to improve health- and skill-related components of physical fitness (e.g., muscle power, balance, speed) in different cohorts (e.g., youth, adults, seniors) (Kraemer et al., 2002). Studies examining the effects of resistance training (e.g., power training, heavy-resistance training) on measures of muscle strength, power, and balance revealed significant improvements in maximal isometric strength (9%), rate of force development (40%), and/or postural sway (20–30%) after 4 weeks of training in healthy adults aged 21–26 years (Bruhn et al., 2004; Gruber et al., 2007). Further, Cormie et al. (2010b) demonstrated that 10 weeks of power training with (un-)loaded jump squats enhanced jump (16%) and sprint performances (3–7%) in resistance-trained males compared with a passive control. Additionally, recent systematic reviews recommended that resistance and power training result in improvements in measures of sprint performances (Bolger et al., 2015; Rumpf et al., 2016).

To maximize the benefits of resistance/power training, the training principles progressive overload, training specificity, and variation should be acknowledged (Kraemer et al., 2002). For instance the principle of training specificity denotes that exercises must attempt to closely mimic the demands of the respective activity (e.g., muscle action, movement velocity, range of motion; Behm, 1995). In fact, research on training programs using different movement velocities during exercises has demonstrated that the greatest adaptations occur at or near the trained velocities (Behm and Sale, 1993). In accordance with the principle of training specificity, resisted sprint training (RST) protocols have become popular training regimes to specifically improve sprint performance. More specifically, inclined surfaces, weight vests/belts, parachutes, sleds, and/or treadmills were introduced as adequate means to increase the load/ resistance during running (Zafeiridis et al., 2005; Paradisis and Cooke, 2006; Spinks et al., 2007; Alcaraz et al., 2008; Harrison and Bourke, 2009; Ross et al., 2009; Clark et al., 2010; Lockie et al., 2012; Kawamori et al., 2014). Harrison and Bourke (2009) reported that 6 weeks of RST in the form of sled pulling enhanced sprint (i.e., 0–5 m time) and jump performances (i.e., drop jump height) in male rugby players compared with a passive control group (mean age: 21 years). Further, Zafeiridis et al. (2005) examined the effects of 8 weeks of sled pulling RST vs. unloaded sprint training on sprint performance and running kinematics in recreationally trained individuals aged 20 years. Following RST compared with an unloaded sprint training group, improvements were found in sprint velocity and stride frequency during the early time intervals (i.e., 0–10 m, 0–20 m) of a 50 m linear sprint test (2–7%). In contrast, sprint velocity in later time intervals (i.e., 20–40 m, 40–50 m) and stride length were enhanced following unloaded sprint training compared with RST (3–5%). These findings indicate that RST is beneficial particularly during the acceleration phase of linear sprints. Additionally, Ross et al. (2009) examined the impact of a 7-week RST that was conducted on a treadmill vs. heavy-resistance training, and a combined RST and strength training protocol on sprint performance in male athletes with a mean age of 20 years. As a result, these authors reported gains in sprint velocity being higher following RST and combined RST and heavy-resistance training (5–8%) when compared to single heavy-resistance training (2%). However, there is no study available that examined how the effects of RST vs. (traditional) power training (i.e., 30–60% 1-repetition maximum) translate to sprint performance (e.g., sprint velocity) and running kinematics (e.g., step length, ground contact time).

Thus, the purpose of the present study was to examine the effects of RST vs. traditional power training (TPT) on measures of physical fitness (e.g., sprint, change-of-direction speed, jump, and balance performance) in healthy young adults. Based on the relevant literature and with reference to the principle of training specificity (Behm and Sale, 1993; Gruber et al., 2007; Ross et al., 2009; Rumpf et al., 2016), we hypothesized larger improvements in sprint performance and running kinematics (e.g., higher sprint velocity and step length, shorter ground contact time) following RST compared with TPT (i.e., primary outcome measures). In terms of change-of-direction speed, jump, and balance performance (i.e., secondary outcome measures), specificity of RST and TPT is less clear which is why we expected general training-related gains following TPT and RST.

## Methods

### Participants

**Figure 1:**
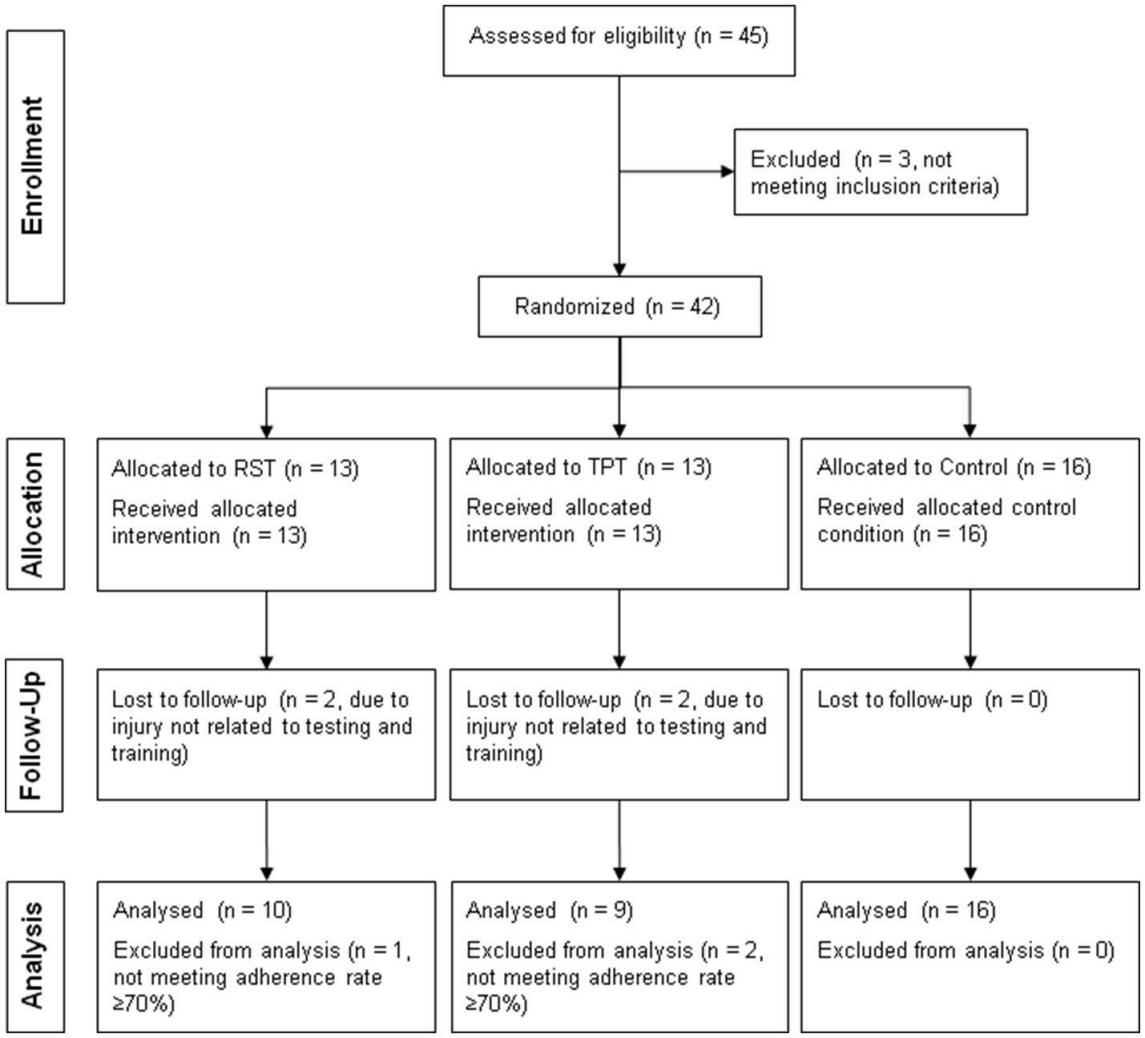
Flowchart of the study design.

In the present study, participants were recruited at the local university campus. Inclusion criteria were (i) age 18–35 years, (ii) sports activity level >60 min/week, (iii) no cardiovascular diseases or acute musculoskeletal, neurological, or orthopedic disorders. With reference to the systematic review article of Rumpf et al. (2016) on the effects of RST on sprint performances, an a priori power analysis with a type I error rate of 0.05 and 80% statistical power was computed. The analysis indicated that 30 participants are sufficient to observe a large-sized main effect (Cohen's *d* = 1.4) of group on 20 m sprint performance (e.g., peak sprint velocity). Because of potential dropouts, 45 healthy, young, and physically active students (17 females, 28 males) were finally enrolled in this study. Written informed consent was obtained before the start of the study. Three subjects were not included in the experimental conditions since the level of sports activity was ≤ 60 min/week. Another seven subjects were excluded from the present study (i.e., per-protocol analysis) due to injury (not related to testing or training) or lack of compliance (< 70% adherence rate) (Dalager et al., 2017). Finally, 35 healthy and physically active participants (12 females, 23 males) were included in the analysis. Male and female participants were randomly assigned to one of two intervention groups (i.e., RST or TPT) or a control group using the method of randomly permuted blocks (stratified randomization: males, females) on a publicly accessible website (http://www.randomization.com). Figure 1 shows a flowchart of the study design. Demographic data of the groups are presented in Table 1. All experiments were approved by the local ethics committee and conducted according to the latest version of the declaration of Helsinki.

**Table 1:** Baseline demographic characteristics of the participants.

	**RST (*n* = 10)**	**TPT (*n* = 9)**	**Control (*n* = 16)**
Sex (females/males)	4/6	2/7	6/10
Age (years)	22.6 ± 2.6	23.4 ± 3.2	22.9 ± 2.4
Body height (cm)	176.6 ± 8.7	178.2 ± 9.0	174.9 ± 8.0
Body mass (kg)	73.5 ± 10.7	72.2 ± 9.6	69.7 ± 10.1
Body mass index (kg/m^2^)	23.4 ± 1.9	22.6 ± 1.5	22.6 ± 2.1
Sports activity level (min/week)	363.0 ± 222.9	457.8 ± 278.8	466.9 ± 254.6

*Data represent mean ± standard deviation. RST, resisted sprint training; TPT, traditional power training*.

### Experimental procedure

As our experimental approach, we used a randomized controlled trial design to examine the effects of RST vs. TPT on proxies of physical fitness in healthy young adults. Following the documentation of demographic data, tests for the assessment of primary (i.e., sprint performance) and secondary outcomes (i.e., change of direction speed, jump, and balance performance) were conducted in the university gym and the biomechanics laboratory 5–7 days prior to and following the intervention period. Training lasted 6 weeks for both experimental groups. Before testing, all participants conducted a standardized warm-up procedure. Warm-up lasted ~10 min and consisted of submaximal running, (rope) skipping, and jumping exercises.

### Training programs

Both experimental groups participated in a 6-week supervised lower limb training program with three training sessions per week (i.e., a total of 18 training sessions). Each session lasted between 45–60 min and a 48 h rest was provided between sessions. The RST protocol comprised resisted sprint exercises (i.e., linear, heel-to-butt, knee lift, jumping, lateral shuffle; Table 2) using elastic straps which were attached to a motorized treadmill (h/p/cosmos quasar med 170/65 with Robowalk Expander, h/p/cosmos Sports & Medical GmbH, Nussdorf-Traunstein, Germany; https://www.youtube.com/watch?v=RV5lkam-I10). During exercise, the ends of the elastic straps were connected to the shank and ankle joint. Three to four repetitions were performed per resisted sprint exercise with each repetition lasting 10 s. Two min of rest was provided between repetitions. Progression during RST was ensured by increasing running velocity (relative to maximal 20 m sprint velocity), treadmill elevation (even vs. slope), and/or resistance (level of stiffness of the elastic straps). In terms of TPT, the participants performed 3–5 sets with 10 repetitions of ballistic lower limb exercises (i.e., leg press, leg curl, knee extension, calve raise; Table 2). Training intensity was set at 40% of the individual one-repetition maximum (Kaneko et al., 1983). Participants were instructed “to act as forcefully and rapidly as possible during the concentric phase of the exercise.” The 1-repetition maximum was determined during the first session using a standardized testing protocol (Baechle and Earle, 2008). The inter-set rest interval amounted to 2 min. During TPT, absolute intensity and volume (i.e., number of sets) were progressively increased for each exercise. Subjects' adherence rates were recorded throughout the intervention period. Participants of the control group were asked to maintain their regular physical activity level throughout the study without specifically participating in sprint and/or power training protocols.

**Table 2:** Progression during the 6 weeks of resisted sprint (RST) and traditional power training (TPT).

	**RST**	**TPT**
**EXERCISES**		
	Resisted linear sprint, heel-to-butt, knee lift, jump running, lateral shuffle	Leg press, leg curl, knee extension, calve raise
**PRESCRIPTION**		
*Week 1*	20–50% maximal sprint velocity, 3 kg expanders, 1.5–8% slope, 10 s, 3–4 reps, 2 min rest	40% 1-RM, 3 sets, 10 reps, 2 min rest, maximal movement velocity
*Week 2*	35–65% maximal sprint velocity, 3 kg expanders, 8% slope, 10 s, 3–4 reps, 2 min rest	40% 1-RM, 3 sets, 10 reps, 2 min rest, maximal movement velocity
*Week 3*	35–65% maximal sprint velocity, 3–5 kg expanders, 8% slope, 10 s, 3–4 reps, 2 min rest	40% 1-RM, 4 sets, 10 reps, 2 min rest, maximal movement velocity
*Week 4*	35–65% maximal sprint velocity, 3–5 kg expanders, 8% slope, 10 s, 4 reps, 2 min rest	40% 1-RM, 4 sets, 10 reps, 2 min rest, maximal movement velocity
*Week 5*	40–70% maximal sprint velocity, 3 kg expanders 8% slope, 10 s, 4 reps, 2–2.5 min rest	40% 1-RM, 5 sets, 10 reps, 2 min rest, maximal movement velocity
*Week 6*	40–70% maximal sprint velocity, 5 kg expanders, 8% slope, 10 s, 4 reps, 2-2.5 min rest	40% 1-RM, 5 sets, 10 reps, 2 min rest, maximal movement velocity

*Expanders in the RST group were bilaterally attached to the shank and/or ankle joint. RM, repetition maximum*.

### Assessment of sprint performance

Spatio-temporal running characteristics of a 20-m linear sprint test were measured using an opto-electronic measurement system (OptoJump next, MicroGate, Bolzano, Italy). The OptoJump-System consists of light-transmitting and -receiving bars. With a continuous connection between two bars, any break in the connection is measured and timed (spatial resolution: 0.03 m; sampling frequency: 1,000 Hz). This method demonstrated high discriminant and concurrent validity [intraclass correlation coefficient (ICC) ≥ 0.93] for the assessment of spatiotemporal gait/running parameters in healthy subjects (Lienhard et al., 2013). Two test trials with a resting period of 2 min between sprints were conducted. Peak sprint velocity was assessed with sprint velocity defined as distance covered per time unit during one step. Additionally, step length, ground contact time, and step frequency during sprinting were measured. The best (highest mean velocity) out of two test trials was used for further analysis.

### Assessment of change of direction speed

Change of direction speed was assessed using the T agility test. Acceptable validity (0.42 ≤ *r* ≤ 0.73) and excellent intrasession reliability (ICC = 0.98) were previously reported for this test (Pauole et al., 2000). For this purpose, a figure-T course was created using 4 cones. Subjects were instructed to run and shuffle as fast as possible passing each cone. Thus, subjects had to continuously change direction throughout the testing procedure. Subjects were able to individually start the test. Two test trials were performed. Performance times were recorded to the nearest 0.001 s using 1 double-light barrier of the WITTY system (MicroGate, Bolzano, Italy). Rest between trials amounted to 2 min. The best (least time) out of two test trials was used for further analysis.

### Assessment of jump performance

To assess lower limb muscle power, participants performed maximal vertical countermovement jumps (CMJ) and drop jumps (DJ) on a 3-dimensional force plate (type 9286AA; Kistler®, Winterthur, Switzerland). For the CMJ test (i.e., jump height), excellent test-retest reliability was reported with an ICC value of 0.98 (Markovic et al., 2004). The vertical ground reaction force was sampled at 1,000 Hz. Prior to testing, participants stood in an erect standing position on the force plate, feet shoulder-width apart, and hands akimbo. Jumps were initiated with a countermovement which was immediately followed by a concentric upward movement. In terms of the DJ, participants stood in an erect standing position on a 36 cm box, feet shoulder-width apart, and hands akimbo. Participants were asked to step off the box with their dominant leg, drop down to the force place and land evenly on both feet, keep ground contact time short, and jump-off the ground with a double-leg vertical jump at maximal effort. Three CMJ and DJ test trials were conducted with a resting period of 0.5 min between jumps and 1 min between CMJ and DJ. The best trial in terms of maximal jump height was taken for further data analysis. Jump height was calculated according to the following formula: jump height = 1/8 × g × t2, where g is the acceleration due to gravity and t is the flight time (Prieske et al., 2013). Additionally, we recorded ground contact time and computed the reactive strength index by dividing jump height by ground contact time).

### Assessment of balance performance

The lower quarter Y-balance test was used to assess dynamic balance. High test-retest reliability was reported for the Y-balance test in all three movement directions with ICC values ranging between 0.89 and 0.93 (Plisky et al., 2006). Before the test started, participants' left and right leg length was assessed as the distance from the anterior superior iliac spine to the most distal aspect of the medial malleolus. Further, participants practiced three trials per reach direction on each foot to get familiarized with the testing procedures. All trials were conducted barefooted. The Y-balance test was performed according to the protocol of Plisky et al. (2006). In brief, participants were positioned in single leg stance while reaching as far as possible with the contralateral leg in three different movement directions (i.e., anterior, posteromedial, posterolateral). Participants always started with the right foot placed at the center of a Y-balance test tool and the left leg reaching three times in anterior direction as far as possible, lightly touching the farthest point possible on the line with the most distal part of the reach foot. Afterwards, the left foot was placed at the center of the grid and the right leg maximally reached in anterior direction. Thereafter, the same test procedure was conducted for the posteromedial and the posterolateral reach direction (positioned 135° from the anterior scale). The examiner manually measured the distance from the scale of the tool. According to Filipa et al. (2010), a composite score was calculated and taken as dependent variable for further data analyses using the following formula: composite score = [(maximum anterior reach distance + maximum posteromedial reach distance + maximum posterolateral reach distance)/(leg length × 3)] × 100.

### Statistical analyses

Normal distribution was examined using the Shapiro-Wilk test. For statistical analyses, an analysis of covariance (ANCOVA) with group as between-subject comparator (RST, TPT, control) and baseline data as a covariate was computed. This method has been proposed as the most sufficient statistical approach for the analysis of continuous outcomes in randomized controlled trials (Vickers, 2005). *Post-hoc* tests with the Bonferroni-adjusted α were calculated to identify the comparisons that reached *p* levels of *p* < 0.1 (i.e., one-tailed hypotheses for primary outcome measures) or *p* < 0.05 (i.e., two-tailed hypotheses for secondary outcome measures). Additionally, group-specific repeated measures ANOVAs (time: pre, post) were applied to evaluate within-group pre- to-post performance changes. Effect sizes were calculated by converting partial eta-squared to Cohen's *d* to indicate whether a statistical difference is a difference of practical concern. According to Cohen (1988), the magnitude of effect sizes can be classified as small (0.2 ≤ *d* < 0.5), medium (0.5 ≤ *d* < 0.8), and large (*d* ≥ 0.8). In general, descriptive data are presented as group mean values and standard deviations. More specifically, post-test data are illustrated as baseline adjusted group mean values and standard deviations. Additionally, group specific pre- to post-test changes are presented as group mean values and 90% confidence intervals (CI). All analyses were performed using Statistical Package for Social Sciences (SPSS) version 24.0.

## Results

All subjects of the RST and TPT groups received treatments as allocated and none of the participants reported any training- or test-related injury. Participants' mean attendance rate during training amounted to 82.2% for RST and 83.9% for TPT and corresponded to a mean training frequency of 2.5 sessions/week throughout the entire intervention period. Table 3 presents baseline data of our primary outcome measures. Additionally, Table 4 displays the baseline-adjusted means and standard deviations of the primary and secondary outcomes at post-test.

**Table 3:** Primary and secondary outcome measures at baseline for the resisted sprint training (RST) group, traditional power training (TPT) group, and control group and the respective group differences.

				**Difference**		
	**RST**	**TPT**	**Control**	**RST-TPT**	**RST-Control**	**TPT-Control**
**PRIMARY OUTCOMES**						
Peak sprint velocity [m/s]	7.92 ± 0.86	8.29 ± 0.59	8.10 ± 0.91	−0.37	−0.18	0.19
Step length [cm]	161.1 ± 9.5	170.4 ± 17.8	167.8 ± 14.1	−9.31	−6.71	2.60
Ground contact time [ms]	136.7 ± 14.6	127.6 ± 7.4	134.2 ± 18.0	9.04	2.47	−6.57
Step frequency [1/s]	4.29 ± 0.38	4.29 ± 0.39	4.18 ± 0.35	0	0.11	0.11
**SECONDARY OUTCOMES**						
*T*-test time [s]	11.0 ± 1.2	10.2 ± 0.6	10.4 ± 0.9	0.84	0.61	−0.23
CMJ height [cm]	29.8 ± 7.3	34.5 ± 7.1	34.1 ± 6.3	−4.69	−4.28	0.41
DJ height [cm]	22.3 ± 5.5	28.5 ± 5.5	29.1 ± 6.6	−6.18	−6.86	−0.68
DJ contact time [ms]	212.5 ± 25.2	188.9 ± 18.6	256.8 ± 71.3	23.61	−44.27	−67.88
DJ reactive strength index [m/s]	1.06 ± 0.28	1.51 ± 0.30	1.23 ± 0.50	−0.45	−0.17	0.28
Y-balance CS (right) [%]	110.9 ± 7.3	115.0 ± 9.7	124.1 ± 7.5	−4.19	−13.29	−9.10
Y-balance CS (left) [%]	110.7 ± 6.5	114.8 ± 9.3	124.1 ± 5.2	−4.12	−13.40	−9.28

*Data represent mean ± standard deviation. CMJ, countermovement jump; CS, composite score; DJ, drop jump; RST, resisted sprint training; TPT, traditional power training*.

**Table 4:** Primary and secondary outcome measures at post-test for the resisted sprint training (RST) group, traditional power training (TPT) group, and control group.

	**RST**	**TPT**	**Control**	***p*-value (effect size *d*)**
**PRIMARY OUTCOMES**				
Peak sprint velocity [m/s]	8.41 ± 0.43	8.36 ± 0.43	8.06 ± 0.43	0.095 (0.81)
Step length [cm]	167.6 ± 7.2	163.8 ± 7.1	167.3 ± 7.0	0.438 (0.47)
Ground contact time [ms]	125.5 ± 7.2	128.4 ± 7.3	133.2 ± 7.1	0.031 (1.00)
Step frequency [1/s]	4.30 ± 0.16	4.35 ± 0.16	4.22 ± 0.16	0.126 (0.76)
**SECONDARY OUTCOMES**				
T-test time [s]	10.3 ± 0.4	10.0 ± 0.4	10.3 ± 0.4	0.136 (0.74)
CMJ height [cm]	33.6 ± 3.0	32.9 ± 2.9	32.6 ± 2.9	0.733 (0.29)
DJ height [cm]	27.5 ± 4.2	28.7 ± 3.8	25.9 ± 3.9	0.201 (0.66)
DJ contact time [ms]	235.1 ± 48.1	216.5 ± 51	219.9 ± 51.5	0.649 (0.34)
DJ reactive strength index [m/s]	1.24 ± 0.31	1.35 ± 0.31	1.29 ± 0.3	0.745 (0.28)
Y-balance CS (right) [%]	116.7 ± 4.9	120.2 ± 4.4	121.3 ± 4.9	0.097 (0.81)
Y-balance CS (left) [%]	117.8 ± 5.6	120.6 ± 4.9	122.4 ± 5.7	0.206 (0.66)

*Data represent baseline-adjusted mean ± standard deviation. CMJ, countermovement jump; CS, composite score; DJ, drop jump; RST, resisted sprint training; TPT, traditional power training*.

### Primary outcomes

**Figure 2:**
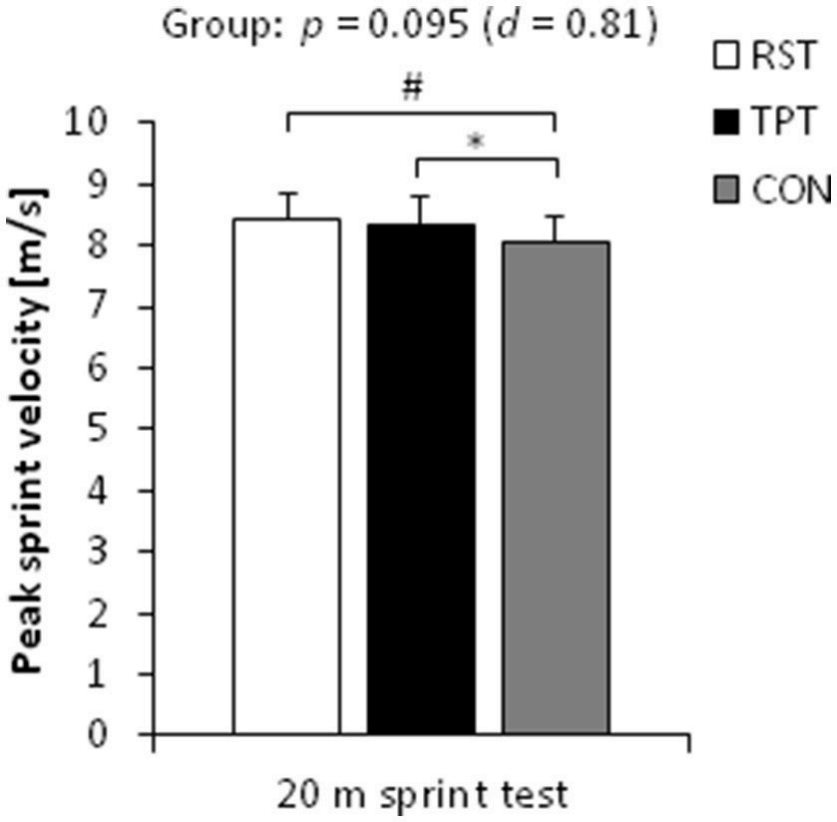
Baseline-adjusted post-test means and standard deviations of peak sprint velocity during the 20 m linear sprint in the resisted sprint training (RST) group, traditional power training (TPT) group, and control (CON) group. ^*^p < 0.05, ^#^*p* < 0.10.

**Figure 3:**
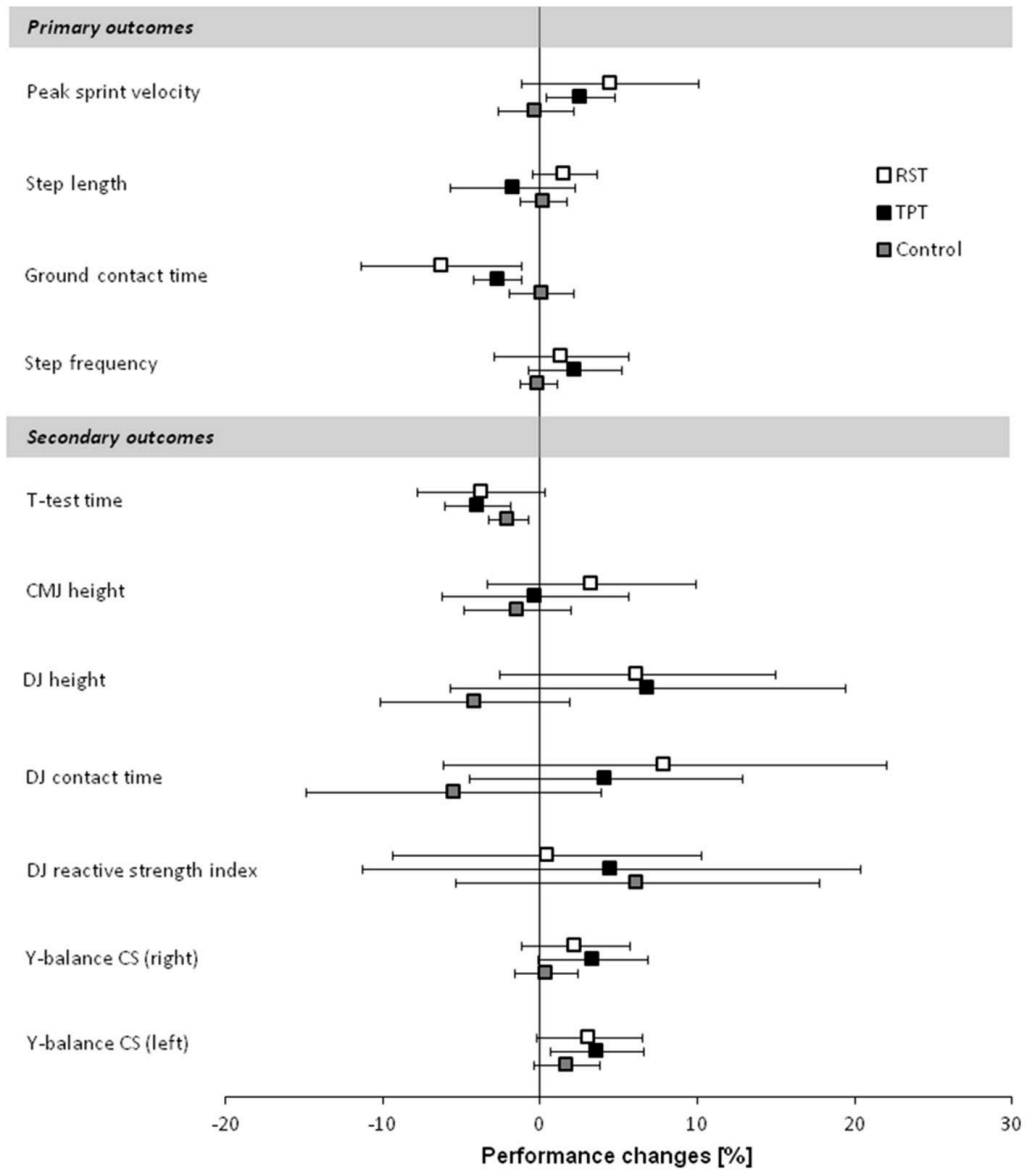
Relative pre- to post-test changes for the resisted sprint training (RST) group, traditional power training (TPT) group, and control group. Data represent mean and 90% confidence interval. CMJ, countermovement jump; CS, composite score; DJ, drop jump; RST, resisted sprint training; TPT, traditional power training.

**Figure 4:**
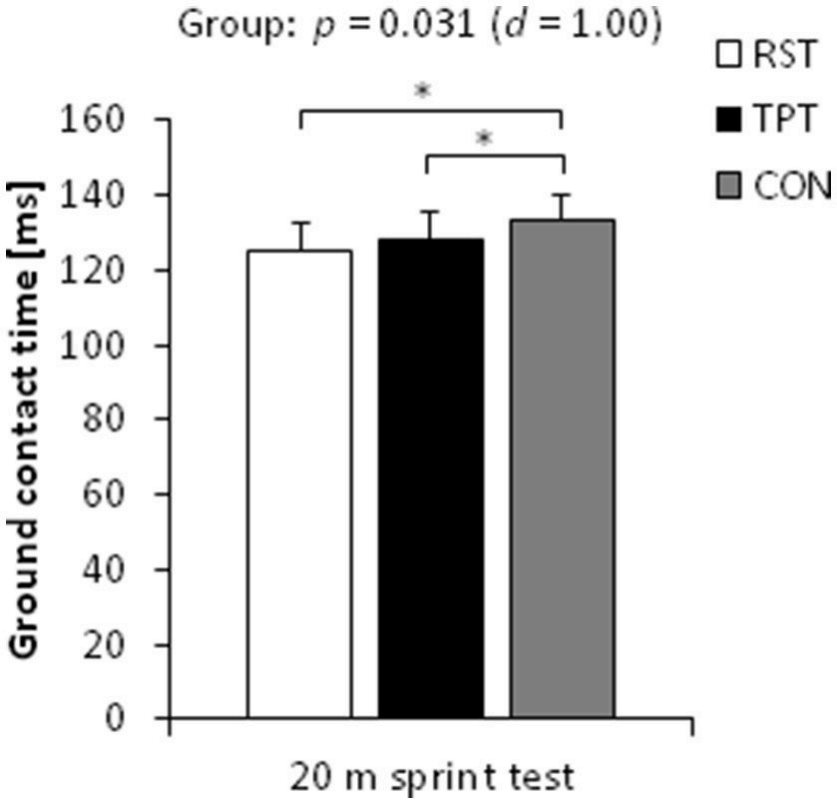
Baseline-adjusted post-test means and standard deviations of ground contact time during the 20 m linear sprint in the resisted sprint training (RST) group, traditional power training (TPT) group, and control (CON) group. ^*^p < 0.05.

In terms of sprint performance, the statistical analysis (one-tailed tests) revealed a large-sized main effect of group for peak sprint velocity (*p* = 0.095, *d* = 0.81). *Post-hoc* tests showed medium- to large-sized effects with higher peak sprint velocities following RST and TPT (4%, *p* ≤ 0.095, 0.69 ≤ *d* ≤ 0.82) compared with the control group. No differences were found between RST and TPT (Figure 2). Large pre-to-post changes were found for RST [4.5%, CI: (−1.1%; 10.1%), *p* = 0.099, *d* = 1.23] and TPT [2.6%, CI: (0.4%; 4.8%), *p* = 0.055, *d* = 1.59] only (Figure 3). Additionally, a large-sized main effect of group was detected for ground contact times during the 20 m sprint test (*p* = 0.031, *d* = 1.00). *Post-hoc* tests revealed medium- to large-sized effects with shorter ground contact times following RST and TPT (4–6%, *p* = 0.044, 0.68 ≤ *d* ≤ 1.09) compared with the control group (Figure 4). Of note, no differences were found between RST and TPT. Large pre-to-post changes were observed for RST [−6.3%, CI: (−11.4%; −1.1%), *p* = 0.058, *d* = 1.45] and TPT [−2.7%, CI: (−4.2%; −1.2%), *p* = 0.010; *d* = 2.36] only (Figure 3). Our ANCOVA analysis detected small- to medium-sized main effects of group for step length and step frequency (*p* > 0.10, 0.47 ≤ *d* ≤ 0.76).

### Secondary outcomes

For the secondary outcome measures (i.e., DJ/CMJ and balance performance, change-of-direction speed), the statistical analysis (two-tailed tests) revealed a large-sized main effect of group (*p* = 0.097; *d* = 0.81) for balance performance (i.e., right leg Y-balance composite score). Finally, small- to medium but less clear effects of group were found for change of direction speed and jump performance (*p* > 0.10, 0.28 ≤ d ≤ 0.74; Table 4 and Figure 3).

## Discussion

To the authors' knowledge, this is the first randomized controlled trial to examine the effects of RST vs. TPT on proxies of physical fitness in healthy, young adults. The main findings of this study were that (i) peak sprint velocity was higher and ground contact times were shorter following RST and TPT compared with control; (ii) RST and TPT induced similar improvements in sprint performance (i.e., peak sprint velocity, ground contact time); and (iii) at post tests, no differences were found between TPT, RST, and control in change-of-direction speed, jump and balance performance.

In order to adhere to the principle of training specificity (Behm and Sale, 1993; Behm, 1995), a variety of RST protocols and training apparatus (e.g., weight vests/belts, parachutes, sleds) were introduced as adequate means to enhance sprint performance. Interestingly, RST programs appear to improve performance and kinematics particularly during the acceleration phase (i.e., 0–20 m) of sprinting in recreationally trained individuals aged 20 years (Zafeiridis et al., 2005). Further, strength-related performance measures (e.g., maximal strength, muscle power) appear to underlie the concept of generality (Hortobagyi et al., 1989). In other words, training of one component of muscle strength (e.g., by means of TPT) may translate to other muscle actions (e.g., power production during running), irrespective of movement velocity. The results of the present study indicate that 20-m sprint performance (i.e., higher peak sprint velocity) and running kinematics (i.e., lower ground contact times) were improved following 6 weeks of RST (4–6%) and TPT (4%) compared with control in healthy young adults. These findings are well in-line with the literature on the effects of RST and TPT programs on sprint performances. For instance, Spinks et al. (2007) examined the effects of 8 weeks of RST using weighted sled pulling vs. unresisted sprint exercises and a passive control on sprint performance in young healthy athletes (i.e., soccer, rugby, American football) with a mean age of 22 years. Following intervention, the RST group produced increases in sprint velocity (6–9%) as quantified by horizontal hip velocity during 15-m sprint bouts but not in the control group. Additionally, Harrison and Bourke (2009) reported that 6 weeks of RST using sled pulling exercises enhanced 0–5 m sprint times in male rugby players aged 21 years compared with a passive control group. Moreover, 7 weeks of RST using a treadmill improved sprint velocity even when compared to strength training only (5% vs. 2%) in former competitive males with a mean age of 20 years (Ross et al., 2009). In terms of TPT, our study confirmed the findings of Delecluse et al. (1995) who examined the effects TPT and strength training on sprint performance in physically active men aged 18–22 years. These authors found that peak sprint velocity was higher (2%) following 9 weeks of TPT compared to a passive control group. In another study, 10 weeks of TPT improved 20-m sprint time (3-4%) in resistance-trained men compared with a passive control group (Cormie et al., 2010b).

From a biomechanical point of view, a recent review article stated that gains in sprint performance following RST may be attributed to improvements in sprint-specific technique, while non-specific speed training such as TPT may improve power production during sprinting (Rumpf et al., 2016). This hypothesis is partly substantiated by reduced ground contact times following RST and TPT as reported in our as well as previous studies (Rimmer and Sleivert, 2000; Spinks et al., 2007). In fact, Spinks et al. (2007) proved that lower ground contact times as a measure of running technique were sufficient to contribute to increases in running velocity following 8 weeks of RST. Additionally, a reduction in ground contact time coupled with an increase in running velocity was discussed to indicate greater power output of the lower limb muscles during sprint running (Spinks et al., 2007). Thus, changes in running technique and/or power production during sprint running following RST and TPT may be responsible for gains in sprint performance in the present study. However, considering that sprint velocity and ground contact time following training were not different between our two intervention groups, RST and TPT appear to be equally effective means to improve measures of sprint performance and running kinematics in healthy and physically active adults.

Interestingly, RST and TPT did not improve change-of-direction speed, jump performance, and balance when compared to the control group. Of note, the literature on the effects of RST and TPT programs on these fitness components revealed inconsistent findings (Spinks et al., 2007; Harrison and Bourke, 2009; Cormie et al., 2010b; Lockie et al., 2012; Balsalobre-Fernández et al., 2013; de Hoyo et al., 2015). For instance, Spinks et al. (2007) reported increases in CMJ height following 8-week of RST. In another study, de Hoyo et al. (2015) demonstrated that 6 weeks of TPT using loaded half squats enhanced CMJ height but not change-of direction speed in the zigzag test in healthy and physically active males. In contrast, Lockie et al. (2012) examined the effects different speed training protocols (e.g., RST, plyometric training) on CMJ and DJ performance (i.e., CMJ/DJ height, DJ ground contact time, DJ reactive strength index) in male field sport athletes with a mean age of 23 years. Following 6 weeks of training, gains in the DJ reactive strength index were found in the RST and the plyometric training group. It was hypothesized that, particularly in the RST group, the lack of changes in DJ/CMJ height may represent the specificity of muscle actions during training. More specifically, power production during RST occurs in horizontal direction, whereas jumping requires vertical power production. Similarly, Balsalobre-Fernández et al. (2013) examined a 10-week TPT using jump squats on jump performance in elite track and field athletes (mean age: 22 years). It was reported that squat jump but not CMJ flight time was enhanced following training. These authors speculated that this finding may be attributed to the applied exercises during training (i.e., ballistic squat jumps) which were not in accordance with the tested muscle actions (i.e., stretch-shortening cycle) during CMJs. Taken these findings into account, we hypothesized that horizontal power development during RST and ballistic muscle actions during TPT may partly explain the lack of transfer to jump performance in the present study.

Further, it has previously been shown that linear sprint performance and change-of-direction speed appear to be specific and independent components of physical fitness (Young et al., 2001; Little and Williams, 2005). For instance, Little and Williams (2005) determined the relationship between sprint performance and change-of-direction speed in professional soccer players aged 18–36 years. The authors reported medium-sized correlation coefficients (0.35 *r* ≤ 0.46) between different performance tests (i.e., 10 m sprint, 20 m flying sprint, zigzag agility test) indicating limited transfer effects from sprint to change-of-direction performance and vice versa. In support of this finding, Young et al. (2001) examined the effects of 6 weeks of sprint training vs. change-of-direction speed training on linear sprint and change-of-direction speed performance in physically active men with a mean age of 24 years. Performance gains were found in the trained task only. It was concluded that sprint training and change-of-direction speed training have specific effects on physical fitness with limited transfer to other components. In line with these observations, enhancements of sprint performance following RST and TPT appeared to be specific to linear sprint performance and did not translate to change-of-direction speed.

This study has some limitations that warrant discussion. First, it should be acknowledged that we did not apply any physiological tests to detect the underlying training-induced adaptive processes responsible for the observed performance changes. More specifically, it remains unknown if RST-related neuromuscular adaptations are different from those observed following TPT (e.g., Cormie et al., 2010a). Second, we used the per-protocol principle for data analysis in this study (i.e., exclusion of participants who did not adhere to the assigned training protocol). In an alternative approach, data could be analyzed regardless of protocol deviations and participants' compliance or withdrawal (i.e., intention-to-treat principle). The inclusion of all randomized participants in the respective groups would give an unbiased estimate of treatment effect and reflect a real clinical situation (Lewis and Machin, 1993).

## Conclusions

In this study, RST was applied using a motorized treadmill and elastic bands. TPT consisted of ballistic strength exercises for the leg and knee extensors, knee and plantarflexors. Six weeks of RST and TPT with each three training sessions per week proved to be safe (i.e., no training-related injuries) and feasible in healthy and physically active young adults. RST and TPT produced similar improvements in 20-m sprint performance compared to a passive control. These findings were accompanied and most likely attributed to the shorter ground contact times following RST and TPT which is indicative of changes in running technique and/or power production during sprinting. Neither RST nor TPT resulted in performance changes in change-of-direction speed, jump and balance performance. Thus, the observed findings from this study indicate that adaptive processes related to TPT and RST are restricted to improvements in sprint performance but do not translate to other components of physical fitness in healthy, young adults. From a practical or coaches' point of view, both training regimes (RST and TPT) can be used for speed development. However, additional training modalities should be included (e.g., plyometric training, balance training) if the goal is to improve change-of-direction speed, jump, and balance performance.

## Author contributions

OP, TK, and UG: Made substantial contributions to conception and design; TK, MA, and EB: Supervised the training sessions and contributed to data collection; OP: Carried out data analysis and interpretation together with TK and UG; OP: Wrote the first draft of the manuscript and all authors were involved in revising it critically for important intellectual content; OP, TK, MA, EB, and UG: Gave final approval of the version to be published and agreed to be accountable for all aspects of the work.

### Conflict of interest statement

The authors declare that the research was conducted in the absence of any commercial or financial relationships that could be construed as a potential conflict of interest.
